# Excessive progesterone impairs mouse decidualization via the Kyn-AhR pathway

**DOI:** 10.3389/fcell.2025.1622998

**Published:** 2025-09-22

**Authors:** Hui-Na Luo, Hong-Yuan Yang, Zai-Mei Wang, Jia-Mei Luo, Tong-Tong Zhang, Zeng-Ming Yang

**Affiliations:** ^1^ College of Veterinary Medicine, South China Agricultural University, Guangzhou, China; ^2^ Key Laboratory of Animal Genetics, Breeding and Reproduction in the Plateau Mountain Region, College of Animal Science, Guizhou University, Guiyang, China

**Keywords:** decidualization, progesterone, IDO1, TDO, KYN, AhR, nucleolin

## Abstract

Progesterone (P_4_) is essential for pregnancy establishment and maintenance. Clinically, P_4_ is widely used to regulate the menstrual cycle, maintain pregnancy, and treat luteal phase deficiency. However, P_4_ administration protocols, particularly regarding routes, dosage, and timing remain poorly defined. Although excessive P_4_ impairs embryo implantation and decidualization in mice, the underlying mechanism remains unclear. Our data show that decidualization in day 8 pregnant mice and artificial decidualization in day 8 pseudopregnant mice are impaired by 4 mg or 8 mg/mouse P_4_. The mRNA levels of *Prl8a2 and Prl3c1,* markers of *in vitro* decidualization are significantly downregulated by 10 or 20 μM P_4_. The uterine fluorescent signal of indoleamine 2,3-dioxygenase 1 (IDO1) and protein levels of tryptophan 2,3-dioxygenase (TDO) are increased after ovariectomized mice are treated with excessive P_4_. Treatment of uterine stromal cells with excessive P_4_ also significantly upregulates the protein levels of IDO1 and TDO, and kynurenine (Kyn) secretion. Epacadostat (IDO1 antagonist) or RU486 (progesterone receptor antagonist) effectively block P_4_-induced Kyn elevation. The mRNA levels of *Prl8a2* and *Prl3c1 and* the protein levels of BMP2 are significantly inhibited by Kyn. The high-dose of P_4_ activates the aryl hydrocarbon receptor (AhR) and its downstream targets CYP1A1 and CYP1B1. Under *in vitro* decidualization, the mRNA levels of *Prl8a2* and *Prl3c1* are inhibited by 2-OH-E_2_ and 4-OH-E_2_, the catalytic products of CYP1A1 and CYP1B1, respectively. CH-223191, a specific AhR antagonist, effectively counteracts the effects of Kyn on *Cyp1a1*, *Cyp1b1*, and *Prl8a2* expression. Additionally, nucleolar size in stromal cells is increased both *in vivo* and *in vitro* following excessive P_4_ treatment. Our findings suggest that excessive P_4_ impairs mouse decidualization via the Kyn-AhR pathway.

## 1 Introduction

Embryo implantation and decidualization are pivotal steps for a successful pregnancy. Decidualization involves the conversion of endometrial fibroblastic stromal cells into specialized decidual cells, which establish a nutrient and immunologically privileged environment for fetal development (Gellersen and Brosens, 2014). Deficiency in embryo implantation and decidualization can lead to adverse pregnancy outcomes, including delayed embryo development, preeclampsia, miscarriage, and preterm birth (Cheng et al., 2023). Ovarian estrogen (E_2_) and progesterone (P_4_) closely regulate this process in mice and humans (Paria et al., 2000). P_4_ is essential for embryo implantation in all studied species (Wetendorf and DeMayo, 2012). In mice, pregnancy maintenance until parturition relies on continuous P_4_ secretion from the corpus luteum (Maurya et al., 2021). P_4_ primarily functions through progesterone receptors (PR), including PR-A and PR-B. Mice lacking both PR-A and PR-B (Pgr^−/−^) exhibit ovarian and uterine defects (Lydon et al., 1995; Lydon et al., 1996).

In clinical practice, P_4_ is widely used for the conservative management of luteal phase deficiency (LPD) and for treating threatened and recurrent abortion (Soules et al., 1977; Daya et al., 1988). LPD is a pregnancy disorder associated with infertility and spontaneous abortion, and the potential etiologies include inadequate P_4_ duration, inadequate P_4_ levels, or endometrial P_4_ resistance (Jones, 1976). Nevertheless, little agreement exists on LPD diagnosis and treatment (Karamardian and Grimes, 1992). Although P_4_ has a significant positive impact on reproductive outcomes in assisted reproduction, the scientific debate remains open regarding P_4_ administration protocols, particularly concerning routes of administration, dosage, timing, and potential interactions with other drugs (Garg et al., 2024). A previous study showed that P_4_ supplementation in natural frozen embryo transfer cycles does not increase the pregnancy rate (Eftekhar et al., 2013). A prospective study also demonstrates that P_4_ has no any significant positive impact on pregnancy outcomes in cases of threatened miscarriage (Boza et al., 2016). Women experiencing recurrent miscarriage exhibit reduced endometrial P_4_ levels. However, it remains unclear whether reduced P_4_ levels can predict or contribute to adverse pregnancy outcomes (McLindon et al., 2023). Concerns exist about progestin use in pregnancy, particularly the potential risk of genital anomalies (e.g., hypospadias in males, female virilization) and non-genital malformations (Carmichael et al., 2005). For clinicians, supplementing P_4_ for all possible LPD patients is an empirical practice. P_4_ as luteal phase support may carry the risk of overconsumption and has adverse effects on pregnancy outcomes. Consequently, it is indispensable to further examine whether excessive P_4_ has any influence on pregnancy outcomes.

Tryptophan (Trp), an essential amino acid, is necessary during pregnancy (Badawy, 2015; Badawy et al., 2016; Hoang et al., 2023; Xue et al., 2023). Trp is mainly metabolized through kynurenine (Kyn) pathway, which is closely associated with various diseases through its metabolites (Stone and Darlington, 2002). Indoleamine-2,3-dioxygenase (IDO) and tryptophan-2,3-dioxygenase (TDO), two key enzymes, regulate the first and rate-limiting step of the Kyn pathway (Austin et al., 2010). IDO and TDO are implicated in various diseases, including inflammation, cancer, diabetes, and mental disorders (Ye et al., 2019). The aryl hydrocarbon receptor (AhR), a ligand-activated transcription factor, is involved in the metabolism of polycyclic aromatic hydrocarbons and estrogens through regulating cytochrome P450 enzymes CYP1A1 and CYP1B1 upon activation by Kyn (Yin et al., 2016; Pacheco and Elizondo, 2023). Furthermore,CYP1A1 and CYP1B1 participate in the metabolism of estrogen and generate 2-hydroxyestradiol (2-OH-E_2_) and 4-hydroxyestradiol (4-OH-E_2_), respectively (Lee et al., 2003). P_4_ regulates TDO2 expression in endometrium and breast tissue, contributing to both normal tissue function and tumor growth (Li et al., 2014; Liu et al., 2020; Hutchinson et al., 2022). Furthermore, activation of the IDO/TDO/Kyn/AhR pathway plays a crucial role in promoting tumor growth (Pacheco and Elizondo, 2023).

In this study, we examined whether excessive P_4_ has any effects on Kyn-AhR pathway during early pregnancy. Our data showed that excessive P_4_ activates Kyn-AhR pathway that suppresses mouse decidualization.

## 2 Materials and methods

### 2.1 Animal treatments

All animal experiments were approved by the Institutional Animal Care and Use Committee of South China Agricultural University. Adult CD1 mice (6–8 weeks old) were maintained in a temperature- and light-regulated environment with a 14 h light/10 h dark photoperiod. Pregnant and pseudopregnant female mice were obtained by mating with fertile or vasectomized male mice, respectively. The day when the vaginal plug was detected was defined as day 1 of pregnancy (D1) or pseudopregnancy.

The P_4_ doses used in this experiment were based on our previous study (Liang et al., 2018). To investigate effects of excessive P_4_ on early pregnancy, pregnant mice were subcutaneously injected with 2, 4, or 8 mg of P_4_ (P0130, Sigma-Aldrich, St. Louis, MO) in 100 μL of sesame oil (S9057, Macklin, Shanghai, China) at 9:00 AM daily from days 3–7. Control mice received 100 μL of sesame oil. On day 8, the mice were sacrificed to collect uteri for further analysis.

To further examine effects of P_4_, ovariectomized mice rested for 2 weeks were subcutaneously injected with 2, 4, or 8 mg of P_4_ in 100 μL of sesame oil for 1, 3, or 7 consecutive days. Control mice received 100 μL of sesame oil. Mice were sacrificed 24 h after the last injection to collect uteri for further analysis.

### 2.2 Artificial decidualization

Artificial decidualization was induced as previously described (Liang et al., 2018). Briefly, on day 4 of pseudopregnancy, 10 μL of sesame oil was injected into one uterine horn to induce decidualization, and the contralateral horn served as a control. Female mice undergoing artificial decidualization were subcutaneously injected with 4 mg P_4_ daily from days 5–7, while controls received 100 μL of sesame oil. On day 8 of pseudopregnancy, mice were sacrificed to collect uteri for further analysis.

### 2.3 Cell isolation, culture and treatments

Mouse endometrial stromal cells were isolated and cultured as previously described (Li et al., 2023a). Briefly, the uteri of day 4 pseudopregnant mice were longitudinally incised and digested with HBSS (PB180321, Procell, Wuhan, China) containing 1% trypsin (0,458, VWR, Radnor, PA) and 6 mg/mL dispase (82,003,500, Sigma-Aldrich, St. Louis, MO). After the uteri were rinsed in HBSS to remove luminal epithelial cells, the remaining tissue was further digested with 0.15 mg/mL collagenase I (2,691,550, Gibco, Grand Island, NY). The collected stromal cells were cultured in DMEM/F12 medium (D2906, Sigma-Aldrich, St. Louis, MO) supplemented with 10% FBS (164,210, Procell, Wuhan, China).

Mouse stromal cells were induced for *in vitro* decidualization using 10 nM E_2_ (HY-B0141, MedChemExpress, NJ, USA) and 1 μM P_4_ as previously described (Chen et al., 2023). The P_4_ doses for the *in vitro* experiments were based on previous studies (Liang et al., 2018; Suthaporn et al., 2021). To investigate the effects of excessive P_4_ on decidualization, stromal cells under *in vitro* decidualization were treated with different doses of P_4_ and analyzed the mRNA levels of *Prl8a2* and *Prl3c1*, markers of mouse *in vitro* decidualization. To examine effects of Kyn on decidualization, stromal cells under *in vitro* decidualization were treated with different concentrations of L-kynurenine (HY-104026; MedChemExpress, NJ, USA).

### 2.4 Kynurenine assay

Kynurenine amount was measured as previously described (Chen et al., 2024a). Briefly, the cultured medium was collected from cultured stromal cells and centrifuged at 5,000×g for 10 min to remove cellular debris. Total 360 μL supernatant was mixed with 180 μL of 30% trichloroacetic acid (TCA; T6399, Sigma-Aldrich, St. Louis, MO) and incubated at 50 °C for 30 min. After the mixture was centrifuged at 3,000 × g for 10 min, the supernatant was thoroughly mixed with an equal volume of Ehrlich reagent (2% p-dimethylaminobenzaldehyde, D109644, Aladdin, Shanghai, China) and incubated for 12–30 min. The absorbance was measured at 492 nm to calculate the concentration using a standard curve of L-kynurenine.

### 2.5 RNA extraction and real-time PCR

qPCR was performed as previously described (Li et al., 2024). Total RNAs were extracted from mouse uterine tissue or mouse stromal cells using TRIzol (AG21101, Accurate Biology, Changsha, China). cDNA was synthesized from RNA using the HiScript II Q RT SuperMix kit (R222-01-AB, Vazyme, Nanjing, China). qPCR was performed using the SYBR Premix (Q311-02-AA, Vazyme, Nanjing, China). The data were analyzed using the 2^−ΔΔ^Ct method and normalized to mouse Rpl7. The primer sequences were listed in Table 1.

**TABLE 1:** Primer sequences used in this study.

Primer sequences	
Mouse -*Cyp1a1*- sense	CAGAAGGTGATGGCAGAG
Mouse -*Cyp1a1*- antisense	ACGGAGGACAGGAATGAA
Mouse -*Cyp1b1*- sense	CTGGACTTGGAGGATGTG
Mouse -*Cyp1b1*- antisense	GCTGGAGAATCGCATTGA
Mouse*-Prl8a2*-sense	AGCCAGAAATCACTGCCACT
Mouse*-Prl8a2*-antisense	TGATCCATGCACCCATAAAA
Mouse*-Prl3c1*-sense	GCCACACGATATGACCGGAA
Mouse*-Prl3c1*-antisense	GGTTTGGCACATCTTGGTGTT
Mouse*-Rpl7*-sense	GCAGATGTACCGCACTGAGATTC
Mouse*-Rpl7*-antisense	ACCTTTGGGCTTACTCCATTGATA

### 2.6 Western blot

Western blot was performed as previously described (Chen et al., 2024b). After tissues or cultured cells were lysed with RIPA (R0010, Solarbio, Beijing, China), the protein concentration was determined by the BCA method (23,225, Thermo Fisher Scientific, Waltham, MA). The samples were separated via SDS-polyacrylamide gel electrophoresis and transferred onto a PVDF membrane (Immobilon®-P, IPVH00010, Millipore, Billerica, MA). After blocked with 5% nonfat milk (A600669, Sangon Biotech, Shanghai, China), the PVDF membranes were incubated with each primary antibody and secondary antibody (1:5,000). The signal was detected using the ECL chemiluminescence kit (Millipore). The primary antibodies utilized in this study include IDO1 (51,851, Cell Signaling Technology, Danvers, MA), TDO (ab259359, Abcam, Cambridge, United Kingdom), BMP2 (A0231, ABclonal, Wuhan, China), SNAIL (3879T, Cell Signaling Technology, Danvers, MA), AhR (A00225-4, Boster, Wuhan, China), CYP1A1 (GTX55582, GeneTex), CYP1B1 (GTX104424, GeneTex), and α-TUBULIN (2144S, Cell Signaling Technology, Danvers, MA), GAPDH (SC-32233, Santa Cruz Biotechnology, Dallas, TX), Histone H3 (ab176842, Abcam, Cambridge, United Kingdom).

### 2.7 Immunofluorescence

Immunofluorescence was performed as previously described (Li et al., 2023b). Briefly, paraffin sections were dewaxed and rehydrated. Antigen retrieval was achieved with citrate buffer (pH 6.0) or Tris/EDTA buffer (pH 9.0). Cell membranes were permeabilized with 0.1% Triton X-100 (T0694, Sangon Biotech, Shanghai, China) in PBS. After non-specific binding was blocked with horse serum (ZLI-9024, ZSGB-BIO, Beijing, China) for 1 h, sections were incubated with each primary antibody overnight at 4 °C and Alexa 488-conjugated secondary antibody (169,549, Jackson ImmunoResearch, West Grove, PA) at 37 °C for 30 min. Nuclei were counterstained with propidium iodide (PI, P4170, Sigma-Aldrich, St. Louis, MO) or 4′,6-diamidino-2-phenylindole (DAPI, D9542, Sigma-Aldrich, St. Louis, MO). Fluorescence signals were captured using a Nikon C2 confocal microscope. The primary antibodies used in this study include IDO1 (66,528-1, Proteintech, Wuhan, China), Phospho-AhR (PA5-36025, Invitrogen, Carlsbad, CA), AhR (A00225-4, Boster, Wuhan, China) and Nucleolin (14,574, Cell Signaling Technology, Danvers, MA).

### 2.8 Cytoplasmic and nuclear extracts

The nuclear and cytoplasmic extractions were conducted as previously described (Deng et al., 2014). Cultured cells were washed twice with pre-chilled PBS, incubated with Buffer B (5 mM EDTA in PBS) on ice for 5 min and scraped off from culture plates. After centrifuged at 1,000 g for 5 min at 4 °C, the pellet was resuspended in Buffer A (10 mM HEPES, 10 mM KCl, 0.1 mM EDTA with fresh added dithiothreitol and phenylmethylsulfonyl fluoride) and shaked at 4 °C for 20 min, mixed with 2.5% Nonidet P-40 and vortexed for 10 s. Following centrifugation at 15,000 g for 5 min at 4 °C, the supernatant was collected as cytoplasmic protein. The remaining pellet was resuspended in Buffer C (20 mM HEPES, 0.4 M NaCl, 1 mM EDTA, freshly added DTT and PMSF), vortexed, and centrifuged at 18,000 g for 5 min at 4 °C, and collected the supernatant as nuclear protein.

### 2.9 Statistical analysis

Data are presented as mean ± standard deviation. The two-tailed Student’s t-test was used to compare two groups. For more than two groups, one-way ANOVA was conducted with *post hoc* tests: LSD (if equal variances were assumed based on Levene’s test) or Games-Howell (if variances were unequal). Statistical significance was set at *P < 0.05, **P < 0.01, and ***P < 0.001.

## 3 Results

### 3.1 Excessive P_4_ impairs decidualization in mice

**FIGURE 1:**
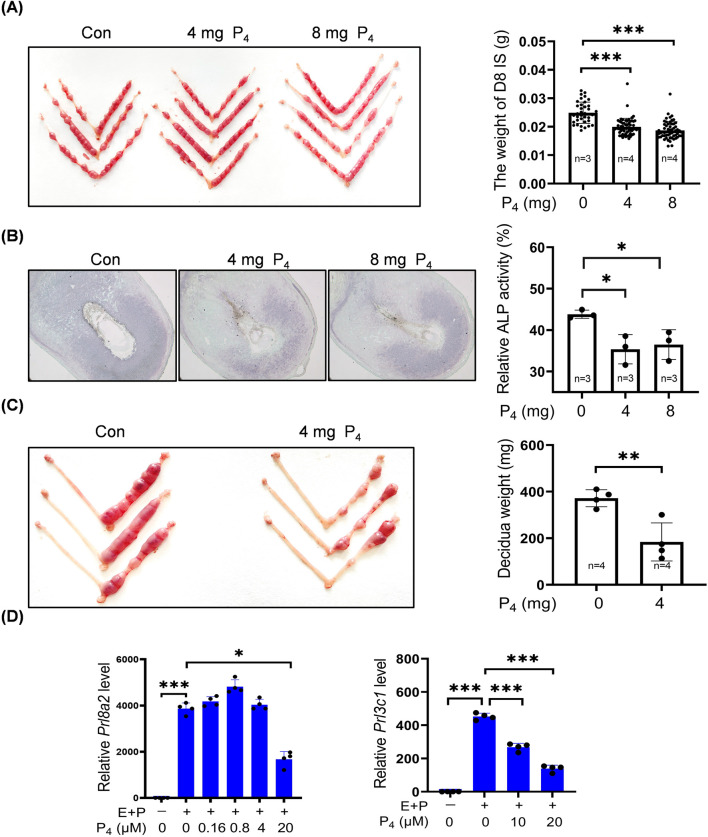
Excessive P_4_ impairs mouse decidualization. **(A)** Representative images and the decidual weights of implantation site on day 8 of pregnancy after pregnant mice were daily treated with P_4_ (4 mg or 8 mg) from days 3–7. **(B)** Alkaline phosphatase staining of day 8 uteri after pregnant mice were treated daily with P_4_ (4 mg or 8 mg) from days 3–7. **(C)** Representative images and the decidual weights of day 8 pseudopregnant uteri after pseudopregnant mice under artificial decidualization were treated daily with 4 mg P_4_ from days 5–7. **(D)** Effects of P_4_ treatment on *Prl8a2* and *Prl3c1* mRNA levels under *in vitro* decidualization for 2 days. The qPCR values were normalized to the *Rpl7* mRNA level. All images are the representative of at least three biologically independent experiments. *, p < 0.05; **, p < 0.01; ***, p < 0.001.

To examine effects of excessive P_4_ on decidualization, pregnant mice were subcutaneously injected with 4 mg or 8 mg of P_4_ in 100 μL sesame oil daily from days 3–7 of pregnancy. Compared with controls, the decidual weight of implantation site on day 8 was significantly reduced by 4 mg or 8 mg P_4_ treatments (Figure 1A). Alkaline phosphatase is a marker of mouse decidualization (Yee and Kennedy, 1988). The staining density of alkaline phosphatase activity in day 8 pregnant uterus was also significantly decreased by 4 mg or 8 mg P_4_ (Figure 1B). Under artificial decidualization, the decidual weight on day 8 pseudopregnant mice was significantly reduced by 4 mg P_4_ treatments from days 5–7 (Figure 1C). *Prl8a2* and *Prl3c1* serve as markers for mouse *in vitro* decidualization (Rasmussen et al., 1997). Under *in vitro* decidualization, *Prl8a2* mRNA was significantly downregulated by 20 μM P_4_, while no significant changes were observed by 0.16, 0.8, or 4 μM P_4_ treatment for 2 days (Figure 1D). Meanwhile, *Prl3c1* mRNA levels were significantly reduced by 10 μM or 20 μM P_4_ (Figure 1D).

### 3.2 P_4_ activates the IDO1/TDO-Kyn pathway

**FIGURE 2:**
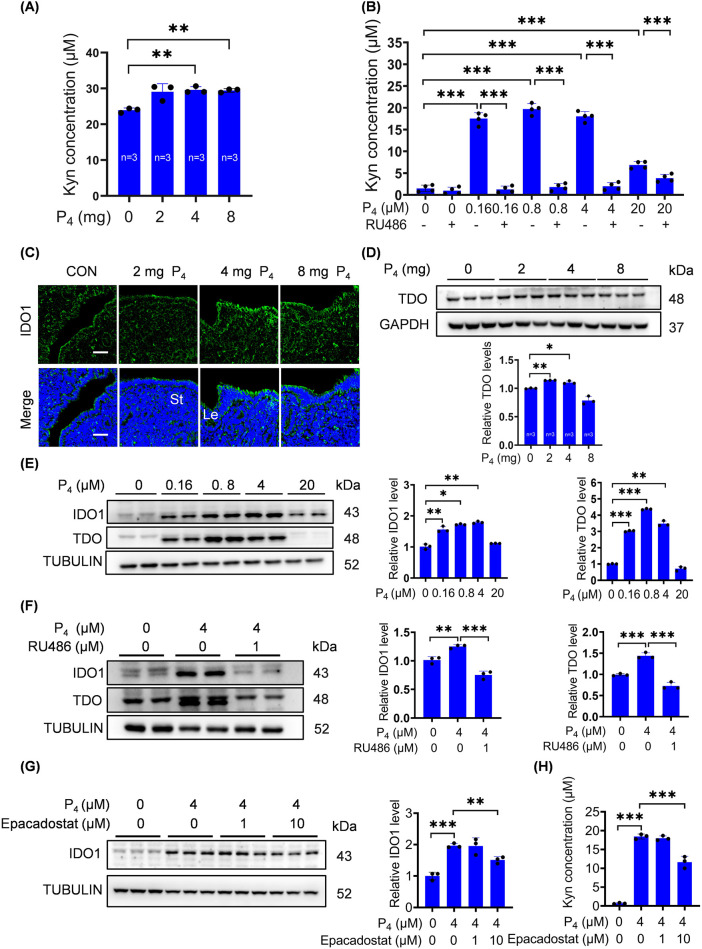
P_4_ activates the IDO1/TDO-Kyn pathway. **(A)** Kyn levels in uterine tissues after ovariectomized mice were subcutaneously injected with 2 mg, 4 mg, or 8 mg P_4_ per mouse for 7 consecutive days. **(B)** Kyn levels in culture medium after stromal cells were treated with P_4_ with or without RU486 for 2 days. **(C)** Uterine IDO1 immunofluorescence after ovariectomized mice were treated with P_4_ (2 mg, 4 mg, 8 mg) for 24 h. Nuclei were counter-stained with DAPI. Le, luminal epithelia; St, stroma. Scale bar, 50 μm. n = 3 mice per group. **(D)** Uterine TDO protein levels after ovariectomized mice were treated with P_4_ for 24 h. **(E)** Western blot analysis of IDO1 and TDO protein levels in stromal cells treated with P_4_ for 3 days. **(F)** IDO1 and TDO protein levels in stromal cells treated with 4 μM P_4_ with or without RU486 for 2 days. **(G)** IDO1 protein levels in stromal cells treated with 4 μM P_4_ with or without Epacadostat for 2 days. **(H)** Kyn levels in the culture medium after stromal cells were treated with 4 μM P_4_ with or without Epacadostat for 2 days. All images are the representative of at least three biologically independent experiments. *, p < 0.05; **, p < 0.01; ***, p < 0.001.

Trp is crucial during pregnancy and mainly metabolized via Kyn pathway (Badawy, 2015; Badawy et al., 2016; Hoang et al., 2023; Xue et al., 2023). TDO, IDO1 and IDO2 are the key rate-limiting enzymes in Kyn pathway and essential for pregnancy (Munn et al., 1998). Because excess P_4_ is detrimental for pregnancy, we wondered whether Kyn pathway was affected by excess P_4_. When ovariectomized mice were treated with 4 mg or 8 mg P_4_ for 7 days, uterine Kyn levels were significantly increased (Figure 2A). IDO1 immunofluorescence signals in the uterine luminal epithelium were clearly increased after ovariectomized mice were treated with 4 mg or 8 mg P_4_ for 24 h, while 2 mg P_4_ had no obvious effect (Figure 2C). Uterine TDO protein levels were also upregulated by 2 mg or 4 mg P_4_, but not by 8 mg P_4_ (Figure 2D).

After stromal cells were treated with 0.16, 0.8, 4, or 20 μM P_4_ for 2 days, Kyn secretion was significantly increased, which was abrogated by RU486, an antagonist of progesterone receptor (Figure 2B). IDO1 and TDO protein levels were also significantly increased after stromal cells were treated with 0.16, 0.8, or 4 μM P_4_ for 3 days (Figure 2E). P_4_-induced increases in IDO1 and TDO protein levels were blocked by RU486 treatments (Figure 2F). Epacadostat, a selective inhibitor of IDO1, effectively suppressed P_4_-induced increases in IDO1 protein levels and Kyn secretion (Figures 2G,H).

### 3.3 Kyn impairs decidualization of mouse stromal cells and activates AhR

**FIGURE 3:**
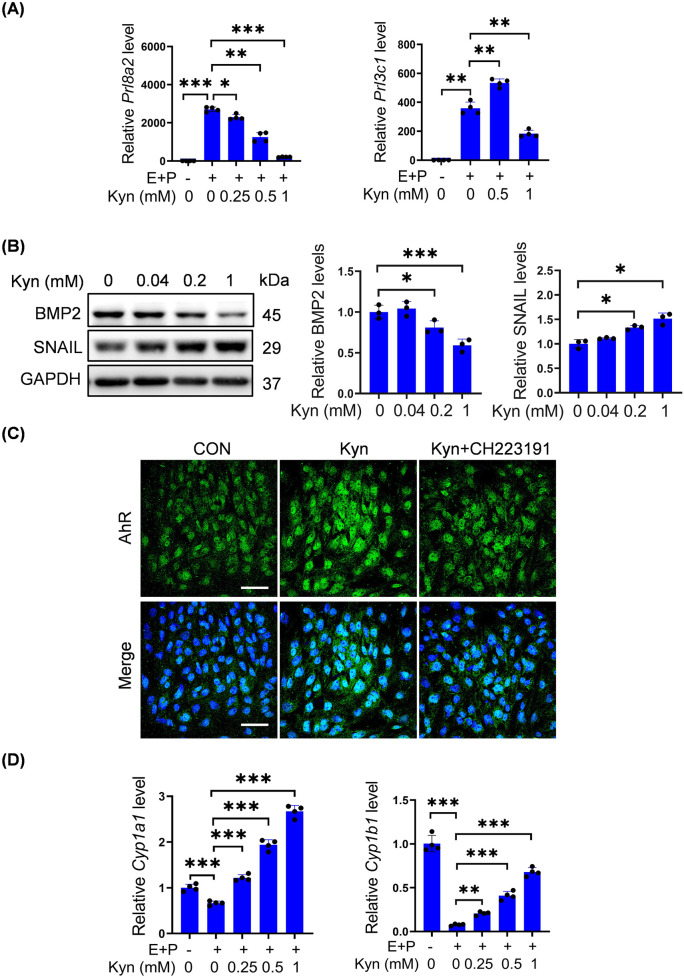
Kyn impairs decidualization of mouse stromal cells and activates AhR. **(A)** *Prl8a2* and *Prl3c1* mRNA levels after stromal cells were treated with Kyn for 2 days under *in vitro* decidualization. **(B)** Western blot analysis and quantification of BMP2 and SNAIL protein levels in stromal cells treated with Kyn for 2 days. **(C)** AhR fluorescence in stromal cells treated with 1 mM Kyn with or without 10 μM CH223191 for 24 h. Nuclei were counter-stained with DAPI. Scale bar, 50 μm. **(D)** The mRNA levels of *Cyp1a1* and *Cyp1b1* after stromal cells were treated with Kyn for 2 days under *in vitro* decidualization. All images are the representative of at least three biologically independent experiments. *, p < 0.05; **, p < 0.01; ***, p < 0.001.

Because high-dose P_4_ increases Kyn levels, we explored whether Kyn had any effects on decidualization. Under *in vitro* decidualization, *Prl8a2* mRNA levels were significantly downregulated in a dose-dependent manner by 0.25, 0.5, or 1 mM Kyn (Figure 3A). Meanwhile, *Prl3c1* mRNA levels were upregulated by 0.5 mM Kyn, but downregulated by 1 mM Kyn (Figure 3A). BMP2 is essential for decidualization (Wang and Dey, 2006). BMP2 protein levels were downregulated after stromal cells were treated with 0.2, or 1 mM Kyn, whereas 0.04 mM Kyn had no detectable change on BMP2 protein levels for 2 days (Figure 3B). SNAIL, a key player during the epithelial-mesenchymal transition, is decreased during decidualization (Zhang et al., 2013; Serrano-Gomez et al., 2016). SNAIL protein levels were significantly upregulated after stromal cells were treated with 0.2, or 1 mM Kyn rather than 0.04 mM Kyn for 2 days (Figure 3B).

Kyn is an effective AhR agonist (DiNatale et al., 2010). Treatment of stromal cells with 1 mM Kyn increased the fluorescence intensity of nuclear AhR, which was abrogated by CH-223191, a specific AhR antagonist (Figure 3C). CYP1A1 and CYP1B1 are downstream targets of AhR (Denison and Whitlock, 1995; Nebert and Dalton, 2006; MacPherson et al., 2013). Under *in vitro* decidualization, *Cyp1a1* and *Cyp1b1* mRNA levels were significantly downregulated, but upregulated in a dose-dependent manner by 0.25, 0.5, or 1 mM Kyn (Figure 3D).

### 3.4 P_4_ activates the AhR-CYP1A1/CYP1B1 signaling pathway

**FIGURE 4:**
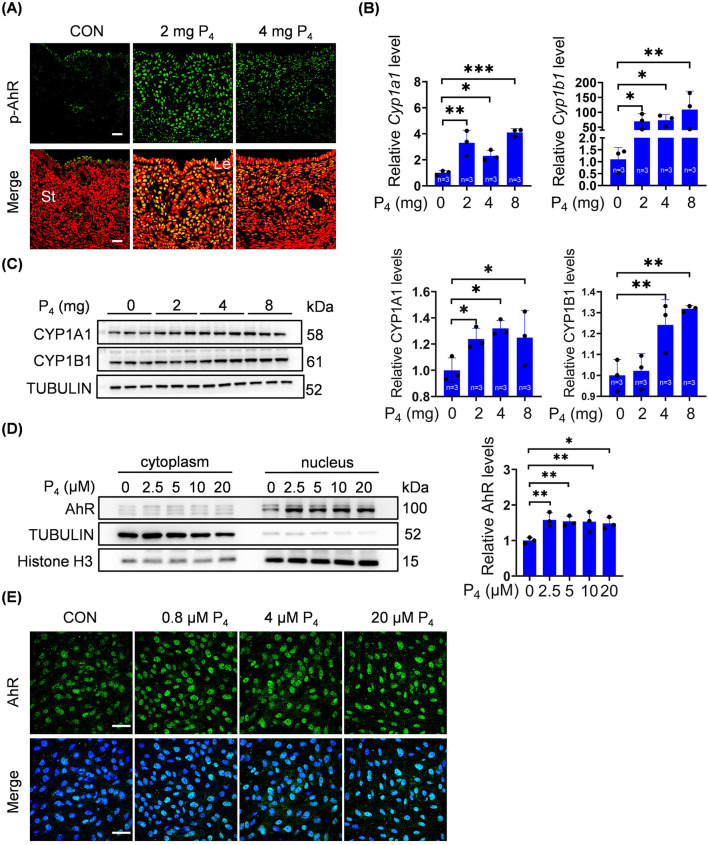
P_4_ activates AhR pathway. **(A)** Uterine phosphorylated AhR immunofluorescence after ovariectomized mice were treated with 2 mg or 4 mg P_4_ for 7 days. Nuclei were counter-stained with PI. Le, luminal epithelia; St, stroma. Scale bar, 20 μm. n = 3 mice per group. **(B)** Uterine mRNA levels of *Cyp1a1* and *Cyp1b1* after ovariectomized mice were treated with 2, 4 or 8 mg P_4_ for 7 days. **(C)** Western blot analysis and quantification of uterine CYP1A1 (3 days injection) and CYP1B1 (7 days injection) protein levels after ovariectomized mice were treated with 2, 4 or 8 mg P_4_. **(D)** Western blot analysis of AhR protein level in nuclear and cytoplasmic fractions, and quantification of AhR in nuclear fractions after stromal cells were treated with P_4_ for 48 h **(E)** AhR immunofluorescence in stromal cells treated with 0.8, 4, or 20 μM P_4_ for 48 h. Nuclei were counterstained with DAPI. Scale bar: 50 μm. All images are the representative of at least three biologically independent experiments. *, p < 0.05; **, p < 0.01; ***, p < 0.001.

We further explored whether excessive P_4_ could directly activate the AhR pathway. When ovariectomized mice were treated with 2 or 4 mg P_4_, p-AhR immunofluorescence in stromal cells was enhanced (Figure 4A). The mRNA levels of *Cyp1a1* and *Cyp1b1* were significantly increased after ovariectomized mice were treated with 2, 4, or 8 mg P_4_ for 7 days (Figure 4B). Furthermore, CYP1A1 and CYP1B1 protein levels in uterine tissues of ovariectomized mice significantly increased after 4 mg or 8 mg P_4_ treatment (Figure 4C). After stromal cells were treated with 2.5, 5, 10, or 20 μM P_4_ for 2 days, nuclear AhR protein levels were clearly elevated (Figure 4D). In addition, nuclear AhR fluorescence in stromal cells was enhanced after treatment with 0.8, 4, or 20 μM P_4_ for 48 h (Figure 4E).

### 3.5 Kyn inhibits stromal decidualization through activating AhR

**FIGURE 5:**
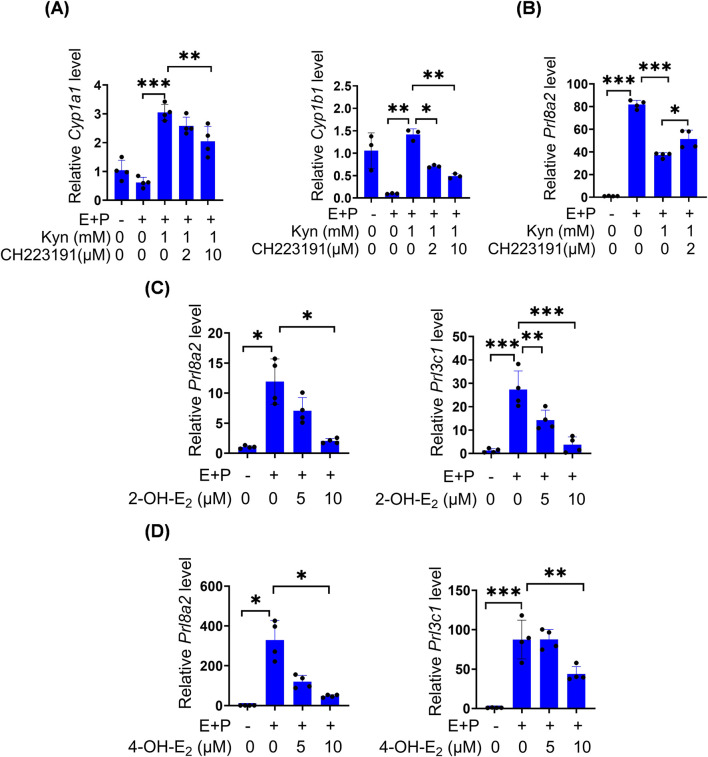
Kyn inhibits mouse stromal cell decidualization through activating AhR. **(A)** The mRNA levels of *Cyp1a1* and *Cyp1b1 after* stromal cells under *in vitro* decidualization were treated with Kyn for 48 h with or without AhR inhibitor CH223191. **(B)** Prl8a2 mRNA level after stromal cells under *in vitro* decidualization were treated with Kyn for 24 h with or without CH223191. **(C)** The mRNA levels of *Prl8a2* and *Prl3c1* after stromal cells were treated with 2-OH-E_2_ for 12 h under *in vitro* decidualization. **(D)** The mRNA levels of *Prl8a2* and *Prl3c1* after stromal cells were treated with 4-OH-E_2_ for 24 h under *in vitro* decidualization. All images are the representative of at least three biologically independent experiments. *, p < 0.05; **, p < 0.01; ***, p < 0.001.

Under *in vitro* decidualization, Kyn significantly suppressed *Prl8a2* mRNA levels, but upregulated *Cyp1a1* and *Cyp1b1* mRNA levels, which were reversed by CH-223191, a specific AhR antagonist (Figures 5A,B). CYP1A1 and CYP1B1 are cytochrome P450 enzymes that catalyze the formation of non-toxic 2-OH-E_2_ and genotoxic 4-OH-E_2_ from E_2_ (Mao et al., 2023). Under *in vitro* decidualization, *Prl8a2* and *Prl3c1* mRNA levels were downregulated by 10 μM 2-OH-E_2_ and 10 μM 4-OH-E_2_, respectively (Figures 5C,D).

### 3.6 Effects of excessive P_4_ on nucleolus

**FIGURE 6:**
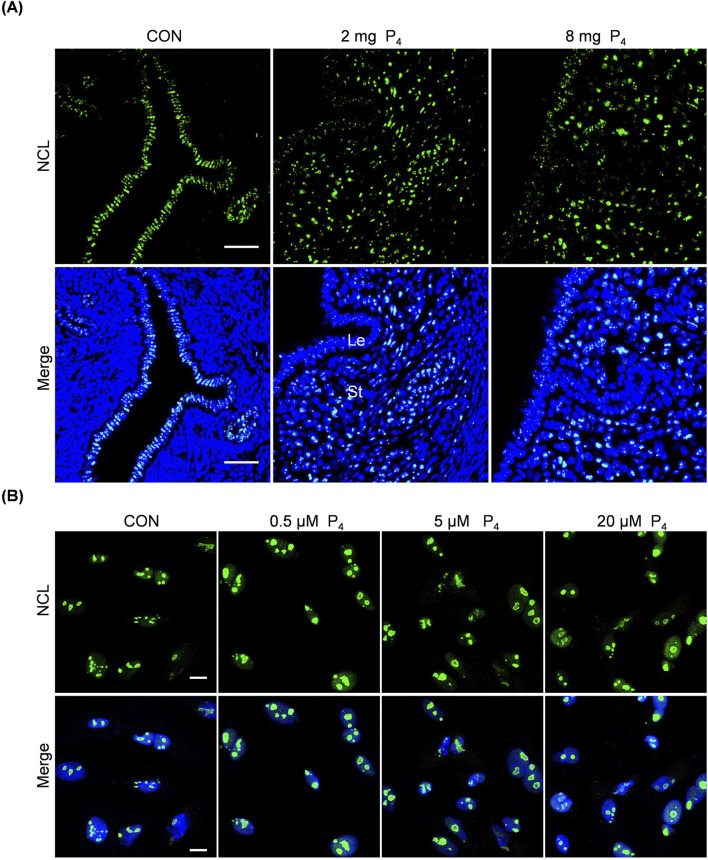
Effects of excessive P_4_ on nucleolus. **(A)** Uterine NCL immunofluorescence after ovariectomized mice were subcutaneously injected with 2 or 8 mg P_4_ for 7 days. Nuclei were counter-stained with DAPI. Le, luminal epithelia; St, stroma. Scale bar, 50 μm. n = 3 mice per group. **(B)** NCL immunofluorescence after stromal cells were treated with 0.5,5 or 20 μM P_4_ for 24 h. Nuclei were counter-stained with DAPI. Scale bar, 20 μm. All images are the representative of at least three biologically independent experiments.

The nucleolus plays a crucial role in ribosome biogenesis. The morphology, size, and activity of nucleolus are closely linked, exhibiting diverse reorganization patterns under stress (Yang et al., 2018). AhR modulates nucleolar activity and enhances protein synthesis (Lafita-Navarro et al., 2018). Given that excess P_4_ was detrimental to pregnancy and could activate the AhR pathway, we investigated excess P_4_ effects on nucleoleus. Nucleolin (NCL), constituting approximately 10% of total nucleolar protein, serves as a nucleolar marker (Lo et al., 2006). After ovariectomized mice were subcutaneously injected with 2 mg or 8 mg P_4_ for 7 days, the size and NCL intensity of nucleolus in the uterine stromal cells were obviously increased, while there were no clear changes for NCL immunofluorescence in luminal and glandular epithelium (Figure 6A). When stromal cells were treated with 0.5, 5, or 20 μM P_4_ for 24 h, the size of nucleolar NCL immunofluorescence was also increased (Figure 6B).

## 4 Discussion

P_4_ is essential for establishing and maintaining pregnancy (Bhurke et al., 2016). However, the potential adverse effects of excessive P_4_ on pregnancy outcomes are frequently overlooked. In this study, we found that excessive P_4_ impaired mouse decidualization both *in vivo* and *in vitro*, potentially through changing tryptophan metabolism and activating AhR pathway.

Numerous studies have reported that excessive P_4_ adversely affects pregnancy outcomes. High P_4_ exposure from the end of menstruation to oocyte maturation is associated with a decreased probability of pregnancy (Kyrou et al., 2011). P_4_ levels ≥1.7 ng/mL before oocyte retrieval significantly reduce endometrial receptivity (Liu et al., 2015). Endometrial gene expression profiles are altered when P_4_ levels exceed 1.5 ng/mL at the end of the follicular phase (Labarta et al., 2011). Elevated P_4_ levels on the day of hCG administration during initial fresh cycles correlate with poor pregnancy outcomes in fresh embryo transfers but not in subsequent frozen-thawed embryo transfers (Venetis et al., 2013). Our previous study also demonstrated that excessive P_4_ impairs mouse embryo implantation and decidualization (Liang et al., 2018).

Trp, an essential amino acid for protein biosynthesis and a precursor of serotonin, has been detected in the ovary, uterus, fallopian tubes, placenta, and ovarian follicular fluid (Doherty et al., 2011; Li et al., 2014). During pregnancy, Trp enhances maternal and fetal protein synthesis, participates in 5-hydroxytryptamine synthesis, provides neuroprotection through kynurenic acid, and suppresses fetal rejection reactions (Xu et al., 2017). Excess Trp must be metabolized early in pregnancy to avoid adverse effects. In mammals, over 95% of free Trp is metabolized through the Kyn pathway, which is closely linked to pregnancy (Stone and Darlington, 2002). Plasma and uterine Trp levels decrease, while Kyn levels increase in human, mouse, and cattle pregnancy (Minatogawa et al., 2003; Schrocksnadel et al., 2006; Groebner et al., 2011). High levels of Trp in culture media inhibit embryo development to the blastocyst stage *in vitro* (McKiernan et al., 1995). Dynamic Trp metabolism serves as a regulatory mechanism to control oxidative stress during pregnancy (Xu et al., 2017). Our previous study demonstrated that Trp deficiency in feed impairs mouse decidualization via the Kyn pathway (Chen et al., 2024a).

IDO1/2 and TDO2 are key rate-limiting enzymes in the Kyn pathway of Trp metabolism (Campesato et al., 2020). IDO1 and TDO2 are intimately associated with the decidualization process (Suzuki et al., 2001; Kudo et al., 2004). IDO1 in mouse placenta is important for preventing the immune rejection of fetal allografts (Sedlmayr et al., 2014). TDO2 can facilitate decidualization in mice (Tatsumi et al., 2000; Li et al., 2014), whereas overexpression of both IDO1 and IDO2 inhibits mouse *in vitro* decidualization (Li et al., 2015a; Li et al., 2015b). IDO1 is possibly involved in endometriosis pathogenesis (Mei et al., 2012). In this study, treatment with excessive P_4_ led to upregulation of IDO1 and TDO protein levels and increased Kyn levels in the mouse uterus and cultured stromal cells. Additionally, high Kyn concentrations inhibited mouse *in vitro* decidualization, suggesting that excessive P_4_ may impair decidualization by activating IDO1 and TDO. P_4_ is able to stimulate IDO1 and IDO2 expression in mouse uterine stromal cells (Li et al., 2015a; Li et al., 2015b). TDO expression is induced by decidualization (Tatsumi et al., 2000). Based on these evidences, it seems that overactivated IDO1 should be detrimental for decidualization.

Kyn, as an endogenous ligand of AhR, activates AhR in mouse stromal cells and induced the expression of downstream genes CYP1A1 and CYP1B1 in our study. AhR is essential for ovarian function, optimizing the fertilization environment, nurturing embryos, maintaining pregnancy, and regulating reproductive lifespan and fertility (Hernandez-Ochoa et al., 2009). AhR is expressed in the pre-implantation mouse uterus (Kitajima et al., 2004). AhR mediates the reproductive toxicity induced by polychlorinated biphenyl congener 126 in rats (Klenov et al., 2021). In early pregnancy, Kyn-AhR enhances NK cell cytotoxicity, contributing to recurrent spontaneous abortion (Yang et al., 2021). Additionally, activation of the Trp/Kyn/AhR pathway promotes the growth of uterine leiomyomas (Zuberi et al., 2023). In our study, AhR was also activated by excessive P_4_, suggesting that overactivated AhR suppresses decidualization.

CYP1A1 and CYP1B1, members of the cytochrome P450 enzyme family, catalyze the formation of 2-OH-E_2_ and 4-OH-E_2_ from E_2_, respectively (Hanna et al., 2000; Lee et al., 2003). CYP1B1 is highly expressed in E_2_ target tissues such as breast, ovary, and uterus (Hakkola et al., 1997). 4-OH-E_2_ generates free radicals through redox cycling with semiquinone and quinone forms, leading to cellular damage and contributing to breast and endometrial cancer development (Tsuchiya et al., 2005). During mouse delayed implantation, 2-OH-E_2_ and 4-OH-E_2_ show no difference in inducing implantation compared to E_2_ (Hoversland et al., 1982). However, in rats, 4-OH-E_2_ is less effective than E_2_ but more effective than 2-OH-E_2_ in initiating implantation (Kantor et al., 1985). Our results demonstrated that both 2-OH-E_2_ and 4-OH-E_2_ inhibit stromal cell decidualization.

Furthermore, based on our NCL immunofluorescence, the nucleolar size was obviously increased both in uterine endometrial stromal cells and cultured stromal cells following excessive P_4_ treatment. These findings suggest that excessive P_4_ may affect endometrial function by altering nucleolar structure and function. The nucleolus, a prominent membraneless structure within the nucleus, plays a crucial role in ribosome formation. This complex process encompasses the transcription of ribosomal DNA (rDNA), the processing of ribosomal RNA (rRNA), and the subsequent assembly of rRNA with ribosomal proteins to generate functional ribosomes (Bassler and Hurt, 2019; Lafontaine et al., 2021). Any disruptions during ribosome biogenesis can induce nucleolar stress, which is marked by changes in nucleolar structure and functionality (Lafita-Navarro and Conacci-Sorrell, 2023). Larger and more nucleoli are frequently observed in tumor cells compared to normal cells, making abnormal nucleolar size and number important indicators for cancer prognosis (Derenzini et al., 2000; Lo et al., 2006). AhR regulates nucleolar activity and protein synthesis (Lafita-Navarro et al., 2018). P_4_ and MPA increase Nucleolin protein levels, which is associated with the proliferative potential of the cells (Yokoyama et al., 1998). Future research could further explore how P_4_ affects embryo implantation and decidualization by influencing the expression of nucleolar-associated proteins.

During decidualization, P_4_ classically affects the endometrium via two well-characterized receptors, PR-A and PR-B (Lydon et al., 1996). However, the effects of P_4_ are also mediated by progesterone receptor membrane component 1 (PGRMC1) (Kaluka et al., 2015). PGRMC1 expression is also tightly regulated at the maternal-fetal interface in humans and rodents (Pru and Clark, 2013). Uterine ablation of PGRMC1 leads to reduced fertility in female mice and the development of endometrial cysts (McCallum et al., 2016). Additionally, P_4_ weakly binds to the nuclear glucocorticoid receptor (GR), which may represent a key mechanism underlying its anti-inflammatory effects in reproductive tissues (Shah et al., 2019). Deficiency in uterine GR signaling results in an exaggerated inflammatory response during induced decidualization, including altered immune cell recruitment (Whirledge et al., 2015). Although this study shows that excessive P_4_ disrupts the Kyn-AhR axis during decidualization, it is still possible that excessive P_4_ may impair decidualization through GR signaling or PGRMC1.

## 5 Conclusion

In summary, our results demonstrate that excessive P_4_ impairs mouse decidualization via activating Kyn-AhR pathway, highlighting the potential mechanisms underlying reproductive disorders and adverse pregnancy outcomes associated with abnormal P_4_ levels.

## Data Availability

The original contributions presented in the study are included in the article/supplementary material, further inquiries can be directed to the corresponding author.

## Abbreviations

AhR, Aryl hydrocarbon receptor; E_2_, Estrogen; IDO, Indoleamine 2,3-dioxygenase; Kyn, Kynurenine; LPD, Luteal phase deficiency; NCL, Nucleolin; Prl3c1, Prolactin family 3, subfamily C, member 1; Prl8a2, Prolactin family 8, subfamily A, member 2; Progesterone, P_4_; PR, Progesterone receptor; PI, Propidium iodide; Trp, Tryptophan; TDO, Tryptophan 2,3-dioxygenase; 2-OH-E_2_, 2-hydroxyestradiol; 4-OH-E_2_, 4-hydroxyestradiol; DAPI, 4′,6-diamidino-2-phenylindole.
